# Gene sequence variations of the platelet P2Y12 receptor are associated with coronary artery disease

**DOI:** 10.1186/1471-2350-8-59

**Published:** 2007-09-05

**Authors:** Ugo Cavallari, Elisabetta Trabetti, Giovanni Malerba, Michele Biscuola, Domenico Girelli, Oliviero Olivieri, Nicola Martinelli, Dominick J Angiolillo, Roberto Corrocher, Pier Franco Pignatti

**Affiliations:** 1 Department of Mother and Child and of Biology-Genetics, Section of Biology and Genetics, University of Verona, Verona, Italy; 2 Department of Clinical and Experimental Medicine, University of Verona, Verona, Italy; 3 Division of Cardiology, University of Florida-Shands Jacksonville, FL USA

## Abstract

**Background:**

The platelet P2Y_12 _receptor plays a key role in platelet activation. The H2 haplotype of the P2Y_12 _receptor gene (*P2RY12*) has been found to be associated with maximal aggregation response to adenosine diphosphate (ADP) and with increased risk for peripheral arterial disease. No data are available on its association with coronary artery disease (CAD).

**Methods:**

The H2 haplotype of the *P2RY12 *was determined in 1378 unrelated patients of both sexes selected according to the presence of significant coronary artery disease (CAD group) or having normal coronary angiogram at cardiac catheterization (CAD-free group). Significant coronary artery disease was angiographically determined, and was defined as a greater than 50% visually estimated luminal diameter stenosis in at least one major epicardial coronary artery.

**Results:**

In the studied population 71.9% had CAD (n = 991) and 28.1% had normal coronary angiogram (n = 387). H2 haplotype carriers were more frequent in the CAD group (p = 0.03, OR = 1.36, 95%CI = 1.02–1.82). The H2 haplotype was significantly associated with CAD in non-smokers (p = 0.007, OR = 1.83 95%CI = 1.17–2.87), but not in smokers. The association remained significant after adjustment for other covariates (age, triglycerides, HDL, hypertension, diabetes) by multivariate logistic regression (p = 0.004, OR = 2.32 95%CI = 1.30–4.15).

**Conclusion:**

Gene sequence variations of the P2Y_12 _receptor gene are associated with the presence of significant CAD, particularly in non-smoking individuals.

## Background

Platelets play a key role in the pathophysiology of atherosclerotic disease and its complications [1,2]. Importantly, platelet function varies considerably among individuals and subjects with enhanced platelet reactivity have increased atherothrombotic risk [3]. The adenosine diphosphate (ADP) P2Y_12 _receptor plays a central role in platelet activation [4]. The relevance of this receptor is demonstrated by the clinical benefit obtained in patients when it is blocked by the P2Y_12 _receptor antagonist clopidogrel [5,6]. Recently, Fontana et al. identified two functional haplotypes designated as H1 and H2, tagged by 4 single nucleotide polymorphisms (SNPs) in absolute linkage disequilibrium (i-C139T, i-T744C, i-ins801A, G52T). Importantly, the minor haplotype (H2) was found to be associated with enhanced platelet reactivity suggesting a role for gene sequence variations of the P2Y_12 _receptor in atherothrombotic processes [7]. Notably, the H2 haplotype has also shown to be associated with peripheral arterial disease [8]. However, its association with coronary atherosclerotic disease has still been poorly explored. To investigate whether this H2 haplotype of the P2Y_12 _receptor gene may be associated with coronary artery disease, we studied the distribution of the i-T744C SNP in 1378 unrelated patients, selected according to the presence of significant coronary artery disease (N = 991) or of normal coronary angiogram at cardiac catheterization (N = 387).

## Methods

### Study population

A total of 1945 consecutive subjects undergoing cardiac catheterization at the University Hospital of Verona were recruited from 1996 to 2005. All subjects were unrelated and of Italian origin from the same geographic area in North-Eastern Italy. Further details on the enrolling criteria have been described elsewhere [9,10].

In the present study we used data from 1378 patients, for whom P2RY12 genotypes were available, selected according to the presence of significant coronary artery disease (CAD group) or having normal coronary angiogram at cardiac catheterization (CAD-free group). Significant coronary artery disease was angiographically determined after the first manifestation of symptoms of ischemic heart disease and was defined as a greater than 50% visually estimated luminal diameter stenosis in at least one major epicardial coronary artery. Most of CAD patients were candidate to coronary artery bypass grafting (CABG). Since the primary aim of our selection was to provide an objective and clear-cut definition of the atherosclerotic phenotype, subjects with non significant coronary stenosis (< 50%) were not included in the study. Most of the patients have not been treated with antiplatelet drugs before the recruitment. After CABG, 90% of patients have been treated with aspirin, about 9% with ticlopidina and 1% with indobuphen. No patients had therapy with clopidogrel. Controls (CAD-free group), mainly examined for valvular heart disease, were enrolled providing that they had not only a normal coronary angiogram at cardiac catheterization, but also neither history nor clinical or instrumental evidence of atherosclerosis in vascular districts other than the coronary bed. The angiograms were assessed by two cardiologists unaware that the patients were to be included in the study.

All individuals gave a written consent before entering the study, which was approved by the Ethical Committee of the University Hospital of Verona.

### Genotyping

Individuals were genotyped for the i-T744C SNP (NCBI refSEQ: rs2046934) of the P2Y_12 _receptor gene (*P2RY12*) in order to tag the H1(744T) and H2 (744C) haplotypes [7]. Genomic DNA was prepared from whole blood samples by standard procedures. Genotyping was performed with LightCycler^® ^2.0 System (Roche Applied Science, Mannheim, Germany) by Real-Time PCR assay and analysis of samples melting curves. Primers and Fluorescence Resonance Energy Transfer (FRET) probes were designed using LightCycler^® ^Probe Design Software 2.0 (Roche Applied Science, Mannheim, Germany), as shown in Table 1.

**Table 1:** Primers and probes used to genotype the i-T744C polymorphism of the P2RY12 gene

**Oligonucleotide**	**Sequence**
Forward primer	5'-ATACTGTGACAACATGATTCTTAATCG-3'
Reverse primer	5'-CACAATAGGCAGCTATAATGAAAACT-3'
Sensor probe	5'-TATCTCTGGTGAAATAAAAAGATTACAAACGTCAT-3'Fluorescein
Anchor probe	RED-5'-CAAATTCCCAAGATGTAGATGCCATATAGCATATT-3'-Phosphate

Genotyping assay consisted of four programs, including pre-incubation (95°C × 10 min), amplification (95°C × 10 sec – 58°C × 10 sec – 72°C × 12 sec, 40 cycles, ramp: 20°C/sec), melting-curve analysis (50°C to 75°C, ramp: 0.2°C/sec) and cooling (20°C × 10 sec). Primers and probes concentrations were determined according to standard protocols. Since the i-T744C SNP of the *P2RY12 *gene is a component in total linkage disequilibrium with other 3 SNPs used to tag the H1/H2 haplotype, subjects carriers of the variant C allele are also referred to as H2 haplotype carriers.

In addition to observing the association of a specific genotype presentation (H1/H1; H1/H2; H2/H2) on CAD, considering also the low number of H2/H2 subjects, we compare subjects homozygous for the wild-type allele with subjects carrying the minor allele, according to a dominant model. Therefore, patients were considered as carriers (H1/H2 and H2/H2) and non carriers (H1/H1) of the H2 haplotype.

### Statistical analysis

All the statistical analyses were performed with the R statistical package [11]. Our calculations, based on the marker allele frequency in CEU population derived from the HapMap Project [12], indicate that our sample size is adequate to detect a SNP or haplotype with a modest effect (significance level = 0.05, power = 80%, OR ≥ 1.42).

Distribution of continuous variables in groups was expressed as mean ± standard deviation. Quantitative data were analysed using Student's t-test. Qualitative data were analysed with a χ^2 ^test. Genotype frequencies of different subgroups of patients were compared by contingency tables. Significant difference in genotype distribution were estimated by χ^2^test. Hardy-Weinberg equilibrium was tested in patients with and without CAD by χ^2 ^test with Yates' correction for continuity. Risk adjustment was performed in a multiple forward stepwise logistic regression analysis. Variables included in this model were age, sex, body mass index, glucose, total cholesterol, HDL, LDL, triglycerides, diabetes, smoking habitus, hypertension status.

## Results

All 1378 subjects were successfully genotyped; of these 991 subjects had significant CAD. Demographics of the study population are described in Table 2. As expected, cardiovascular risk factors were more frequently found in subjects with angiographic evidence of significant CAD.

**Table 2:** Demographics of the study population in the CAD and CAD-free groups

	**CAD = 991**	**CAD-free = 387**	**p-value**
Age (yr)	61.38 ± 9.81	58.78 ± 12.3	< 0.001
Males	79.8%	65%	< 0.001
Body Mass Index (kg/m^2^)	26.74 ± 3.54	25.35 ± 3.45	< 0.001
Glucose (mmol/l)	5.84 ± 1.6	5.51 ± 0.93	< 0.001
Total cholesterol (mmol/l)	5.56 ± 1.17	5.44 ± 1.11	n.s.
HDL (mmol/l)	1.19 ± 0.31	1.42 ± 0.42	< 0.001
LDL (mmol/l)	3.73 ± 0.99	3.52 ± 0.93	< 0.01
Triglycerides (mmol/dl)	1.92 ± 1.05	1.48 ± 0.68	< 0.001
Diabetes Mellitus	20.1%	7.2%	< 0.001
Hypertension	65.4%	40.4%	< 0.001
Current smokers	65.6%	41.1%	< 0.001

In the overall patient population, the allele and genotype distribution were as follows: C allele frequency, 12.8%; H1/H1, 1046 (75.9%); H1/H2, 310 (22.5%); H2/H2, 22 (1.6%). Genotype distribution of the i-T744C polymorphism tagging the H1/H2 haplotype in the study population in the CAD and CAD-free groups is shown in Table 3. C allele frequency was 13.47% and 11.24% in CAD and CAD-free subjects, respectively (p > 0.05). Genotype distribution was in Hardy-Weinberg equilibrium. A significant difference was observed in the distribution of the three genotypes between the two groups (p = 0.017). Carriers of the minor haplotype (H1/H2 + H2/H2) were more frequent in patients with significant CAD (p = 0.03, OR = 1.36, 95%CI = 1.02–1.82). Such association was close to significance in males (p = 0.05), but not in females, even if in the two subgroups the H2 haplotype carriers tended to be more frequent among CAD patients (data not shown). The association remained close to significance threshold after adjustment for other covariates (age, sex, BMI, triglycerides, HDL, LDL, hypertension, smoking and diabetes) by multivariate logistic regression (p = 0.05, OR = 1.42, 95%CI = 1.18–1.71).

**Table 3:** Genotype frequency of the i-T744C SNP of the P2RY12 gene in patients with and without significant coronary artery disease

	**CAD**	**CAD-free**	OR (CI 95%) p-value*
**T/T**	737 (74.37%)	309 (79.84%)	
**T/C**	241 (24.32%)	69 (17.83%)	1.36
**C/C**	13 (1.31%)	9 (2.33%)	1.02–1.82
**TC+CC**	254 (25.63%)	78 (20.16%)	0.03

*T/C and C/C genotypes (H1/H2 and H2/H2 haplotypes, respectively) are pooled in the CAD and in the CAD-free group for p-value and OR calculation.

The hypothesis of interaction between the *P2RY12 *gene and variables involved in atherosclerotic risk was then evaluated. Interactions with a p-value ≤ 0.1 were further evaluated through stratification of the studied population for the interacting factor. Only smoking met this criterion (p for interaction = 0.026), consequently genotype distribution of the i-T744C polymorphism of the *P2RY12 *gene was evaluated in smokers and non-smokers as shown in Table 4. Non-smokers carrying the minor haplotype H2 were highly associated with significant CAD (p = 0.007, OR = 1.83, 95%CI = 1.17–2.87). The association remained significant after adjustment for other covariates (age, sex, BMI, triglycerides, HDL, LDL, hypertension and diabetes) by multiple logistic regression (p = 0.004, OR = 2.32, 95%CI = 1.30–4.15) and after adjustment for multiple tests (p = 0.014). No other significant interaction was found between the H2 haplotype of the *P2RY12 *gene and other variables.

**Table 4:** Genotype frequency of the i-T744C SNP of the P2RY12 gene according to smoking habit

	**Smokers (n = 809)**			**Non-smokers (n = 503)**		
	
	**CAD**	**CAD-free**	OR (CI 95%) p-value *	**CAD**	**CAD-free**	OR (CI 95%) p-value *
**T/T**	490 (75.4%)	121 (76.1%)		221 (72.5%)	164 (82.8%)	
**T/C**	150 (23%)	35 (22%)	1.04 (0.69–1.56)	81 (26.5%)	28 (14.2%)	1.83
**C/C**	10 (1.6%)	3 (1.9%)	p = 0.85	3 (1%)	6 (3%)	(1.17–2.87)
**TC+CC**	160 (24.6%)	38 (23.9%)		84 (27.5%)	34 (17.2%)	p = 0.007

*T/C and C/C genotypes (H1/H2 and H2/H2 haplotypes, respectively) are pooled in the CAD and in the CAD-free group for p-value and OR calculation.

## Discussion

The present report is the first to demonstrate an association between the minor H2 haplotype of the *P2RY12 *gene and presence of significant coronary artery disease. Thus the finding of the present study further extends our knowledge on the implications of gene sequence variations of this receptor, a key player in platelet function, on atherothrombosis. In fact, to date this polymorphism has shown to be associated with peripheral arterial disease [8], while its association with coronary artery disease has been poorly explored.

One prior report has assessed the association between this haplotype and long term complications of coronary artery disease (cardiac death, myocardial infarction and refractory angina requiring revascularization) but failed to find any association [13]. It may be argued that this study had a limited statistical power to detect the implications of this polymorphism given the relatively small sample size of the study and the different types of patient population evaluated.

The *P2RY12 *gene is located in the P2 receptor gene cluster on chromosome 3q24-q25. The mechanisms through which these gene sequence variations might lead to an increased atherothrombotic risk are still not well established. Fontana et al. showed that the H2 haplotype of the *P2RY12 *gene is associated with increased platelet function in non-medicated healthy volunteers [7]. Notably, platelet activation plays a leading role in the initiation and development of atherosclerosis as well as in its complications. The seminal findings from Fontana et al have led to the hypothesis that this genetic polymorphism may be responsible for modulation of individual responsiveness to antiplatelet agents.

On the other hand, platelet function studies performed by the same authors as well as other executed in medically treated patients with clinical manifestations of atherosclerotic disease did not show any modulating effects of this genetic polymorphism on individual responsiveness to aspirin or clopidogrel [14-17]. Therefore, despite the key role of P2RY12 receptor on inducing platelet activation, it is likely that genetic polymorphisms of other targets may be more important in modulating aspirin and clopidogrel effects[18]. Moreover, Hetherington et al [19] reported no significant effect of P2RY12 SNPs, including the i-T744C one, on platelet response to ADP in subjects without history of CAD, and an association of a common variant in P2Y1 gene with platelet reactivity.

Atherosclerosis is a multifactorial disease and involves both environmental and genetic factors. Therefore, the low penetrance of a single functional polymorphism may not always lead to a clinical phenotype as this may be obscured by environmental factors. This may explain why the H2 haplotype of *P2RY12 *gene had its strongest association with CAD in non-smoking individuals. Notably, platelets isolated from smokers exhibit increased reactivity as well as spontaneous aggregation [20]. Moreover, nicotine upregulates the expression of P2Y_12 _receptors on vascular cells and megakaryoblasts, and smokers exhibit higher P2Y_12 _expression [21]. Therefore, the presence of genetic determinants leading to increased platelet function profiles may be masked by cigarette smoking. Notably, the association between the H2 haplotype and CAD in non-smokers remained significant even after adjustment for other covariates (age, sex, BMI, triglycerides, HDL, LDL, hypertension, smoking and diabetes) by multivariate logistic regression, suggesting that the P2RY12 gene is an independent risk factor for coronary artery disease in these subjects.

The complex interplay between genetic and environmental factors on the development of atherosclerotic disease explains why large study populations are required to define if a particular functional polymorphism may have effects not only on intermediate phenotypes (i.e. platelet activation), but also on clinical phenotypes (e.g. atherosclerosis). In the present study, as expected, conventional cardiovascular risk factors were more common in patients with CAD. However, it was also demonstrated that the H2 haplotype was more frequently present in patients with CAD. This observation is in keeping with the concept that in multifactorial diseases, genetic polymorphisms influence the risk of disease by determining a different individual susceptibility to environmental risk factors rather than being the cause of the disease process itself.

The primary end-point of the present work was to investigate the association between CAD and the i-T744C SNP of the P2RY12 gene through a case-control study in unrelated individuals. Certainly the study has some limitations, such as the relatively low number of controls and no available data on platelet aggregation. On the other hand, the sample size of our study-population was adequate to detect a mild effect on phenotype by this genetic variant. A strength of this work was the clear-cut definition of CAD phenotype on the basis of coronary angiography, which is of pivotal importance in the analysis of genotype-phenotype correlation. Male and female subgroups showed H2 haplotype carriers more frequently in CAD patients, and it is likely that these observation did not reach the statistical significance due to the small size of each group after stratification.

## Conclusion

This study supports the previously reported association of *P2RY12 *gene with atherosclerosis, giving for the first time genetic association data for the presence of significant coronary artery disease, particularly in non-smoking individuals. Nonetheless, this correlation remains still controversial [7,19] and further studies, possibly with prospective design, are needed to confirm these data.

## Competing interests

Dominick J. Angiolillo is on the speaker bureau and is a consultant for Sanofi-Aventis and Bristol Myers Squibb. The remaining authors report no conflicts.

## Authors' contributions

UC conceived of the study, carried out genotyping and drafted the manuscript. ET designed of the study and participated in its coordination and helped to draft the manuscript. GM performed the statistical analyses. MB carried out genotyping. NM helped in the analysis and interpretation of data. DJA, DG, OO, RC and PFP helped in revising the manuscript critically for intellectual content. All authors read and approved the final manuscript.

## Pre-publication history

The pre-publication history for this paper can be accessed here:


